# Why Did I Consult My Pharmacist about Herbal and Dietary Supplements? An Online Survey Amid the COVID-19 Pandemic in Malaysia

**DOI:** 10.3390/ijerph191710994

**Published:** 2022-09-02

**Authors:** Mohd Shahezwan Abd Wahab, Muhammad Mustaqim Jalani, Khang Wen Goh, Long Chiau Ming, Erwin Martinez Faller

**Affiliations:** 1 Faculty of Pharmacy, Universiti Teknologi MARA (UiTM) Cawangan Selangor, Kampus Puncak Alam, Puncak Alam 42300, Malaysia; 2 Non-Destructive Biomedical and Pharmaceutical Research Centre, Smart Manufacturing Research Institute, Universiti Teknologi MARA (UiTM) Cawangan Selangor, Kampus Puncak Alam, Puncak Alam 42300, Malaysia; 3 Faculty of Data Science and Information Technology, INTI International University, Nilai 71800, Malaysia; 4 PAP Rashidah Sa’adatul Bolkiah Institute of Health Sciences, Universiti Brunei Darussalam, Gadong BE1410, Brunei; 5 Faculty of Pharmacy, San Pedro College, Davao City 8000, Philippines

**Keywords:** herbal and dietary supplements, community pharmacist, community pharmacy, consultation, Malaysia

## Abstract

Herbal and dietary supplements (HDSs) are frequently obtained from community pharmacies, but community pharmacists (CPs) have been underutilized for information regarding them. This study aimed to determine the prevalence of, factors behind, and reasons for consultation with CPs among HDS consumers in Malaysia. A cross-sectional study using an online survey was conducted among conveniently sampled individuals in Malaysia. Reasons for consultation or non-consultation with CPs about HDSs were sought from the respondents. A logistic regression analysis was conducted to determine the predictors of consultation with CPs. Overall, 40.3% (239/593) of participants consulted CPs about the HDSs that they purchased. The participants were predominantly unmarried (362/588, 61.6%) and belonged to the 18–29 age group (332/593, 56%). The multivariate analysis showed that a suburban residential setting was the only significant predictor for consultation with CPs (adjusted odds ratio = 0.390, 95% CI = 0.260–0.583). Respondents who consulted CPs generally agreed that the CPs were the right people to consult on HDSs (mean = 4.37, SD = 0.73). However, their discussion with CPs regarding HDSs mostly revolved around the benefits and directions for use, but little on the potential risks. Meanwhile, most respondents who did not consult CPs agreed that they had never thought of consulting CPs about their HDS use (mean = 3.45, SD = 1.02). The majority of them referred to the Internet (61.3%, 217/354) and social media (59.9%, 212/354) for information about HDSs. The findings from this study show that more efforts are warranted in encouraging consumers to consult CPs about their HDS use and to enhance their awareness of the roles of CPs in ensuring the safe use of HDSs.

## 1. Introduction

Herbal and dietary supplements (HDSs) are widely used in Malaysia [1,2,3] as well as in other countries [4,5,6]. HDS products are readily accessible over the counter with no prescription required to obtain them. Therefore, consumers have the autonomy to use HDSs for their self-care. Among consumers, HDSs are not only used to maintain health, but also for the prevention and treatment of diseases [2,7]. Over the past years, the HDS market has grown progressively due to the increased demand for the products among the public.

In the United States (U.S.), the National Health and Nutrition Examination Survey (NHANES) reported that the prevalence of HDS use among U.S. adults was approximately 50% in 2003–2006 (54%), 2007–2010 (49%), and 2011–2014 (52%). In comparison, the prevalence was only 23% in 1971–1974, and 35% in 1976–1980 [8,9]. Similarly, the prevalence of HDS use in Malaysia has increased considerably over the past few years. The Malaysian Adults Nutrition Survey (MANS) in 2014 reported that 28% and 34% of adults in Malaysia utilized multivitamin–mineral supplements and food supplements, respectively. Of note, the use prevalence of multivitamin–mineral supplements and food supplements in 2013 was 23.9% and 24.8%, respectively [10].

Today, numerous products are available on the market. Notably, the demand for HDSs has increased tremendously following the outbreak of the coronavirus disease (COVID-19) [11], resulting in skyrocketing sales of the products [12]. In a recent survey conducted in Hong Kong during the COVID-19 pandemic, the prevalence of use of herbal products and dietary supplements (DSs) was 19.3% and 25.3%, respectively [13]. In a survey in Saudi Arabia, about one-fifth (22.1%) of the respondents reported using HDSs to prevent COVID-19 [14]. HDSs such as garlic, ginger, black seeds, vitamin C, and honey were reported to be prevalently used by the public during the COVID-19 pandemic [14,15,16,17].

A remarkable interest in HDSs, especially those with antioxidant activities, has been observed in recent years due to their potential preventive effects against oxidative stress, which plays a vital role in the pathogenesis of chronic diseases in the human body. At present, many studies have been conducted to investigate the mechanism and capacity of the antioxidant activity of various natural products [18,19,20,21,22]. The findings from such investigations have prompted the development of various HDSs for human health.

Some reasons that motivate consumers to use HDSs include proactive behavior for self-care [6,23], recommendations from close relatives and colleagues [24], and the influence of advertisements regarding the health claims of HDSs [25]. Studies have reported that consumers perceived HDSs as safer and having fewer side effects than conventional medicines [26,27]. Despite such beliefs, the products can in fact exert pharmacological effects and therefore may cause adverse reactions and interact with conventional medicines [28]. The adverse effects of HDSs and their association with hospitalizations and emergency department visits have been reported elsewhere [29,30]. The concurrent use of HDSs and conventional medicines can potentially alter the bioavailability or efficacy of the latter, which may cause treatment failure or enhance their toxicity. Thus, it is crucial for consumers who are planning to use HDSs to discuss this with health care professionals (HCPs) who can assess the appropriateness and provide advice to ensure the safe use of the products.

HDSs are widely available from community pharmacies, putting community pharmacists (CPs) in an ideal position to ensure that consumers are using the products appropriately and safely [31]. In general, CPs have the knowledge to determine whether self-medication with HDSs is appropriate and whether consumers require medical attention instead [32]. CPs are also trained in pathophysiology and pharmacology, and are able to appraise scientific reports on HDSs and interpret the findings [32]. Therefore, CPs can assist consumers in selecting the most appropriate HDS based on their health needs, and provide them with counselling on the safe use of the products [33]. Additionally, as medication-use experts, CPs can identify problems associated with HDS use (e.g., overdosage, adverse effects, HDS-drug interactions, etc.) and help to solve or prevent these issues [33,34].

Nevertheless, previous studies have shown that CPs are underutilized by consumers for information regarding HDSs [1,3]. For example, in a recent study in Malaysia involving suburban consumers, only about 23% cited pharmacists as their source of HDS information, despite almost 56% obtaining the products from a pharmacy [1]. In another study, 71.8% of urban consumers purchased HDSs from a pharmacy but only about 35% referred to a pharmacist for HDS information [3]. Additionally, multiple studies have reported that the communication on HDSs among consumers and HCPs including pharmacists is generally low [35,36,37]. On the other hand, other studies have suggested that the initiation of conversation by consumers or the act of consulting CPs about HDSs through inquiries could promote consumer–CP discussion around these products [38,39,40]. A conversation or consultation about HDSs initiated by consumers would signal to CPs that their participation in consumer decision-making is being solicited, thus facilitating a healthy consultation session [40,41].

Hence, there is a need to understand the factors and the reasons behind consumers consulting CPs regarding their HDS use. Therefore, the aims of the present study were to determine the prevalence of, factors behind, and reasons for consultation about HDSs with CPs among consumers in Malaysia. The findings from this study can provide useful insights into the consumers’ perceptions about consulting CPs regarding HDSs, and may inform necessary strategies to enhance their engagement with CPs. This may help narrow the gap in communication about HDSs between the consumers and CPs.

## 2. Methodology

### 2.1. Study Design and Setting

This was a cross-sectional survey study conducted over three weeks from 4 to 25 April 2022 involving a convenience sample of individuals in Malaysia. This study was approved by the Research Ethics Committee of *Universiti Teknologi* MARA (UiTM), Malaysia (REC[PH]/014/2022). The study procedures and results are reported according to the Checklist for Reporting Results of Internet E-Surveys (CHERRIES) [42] (Appendix A).

In this study, the HDSs referred to herbal products and DSs that are taken orally in the form of pharmaceutical formulations such as pills, capsules, tablets, or liquids/mixtures. Herbal products are those containing plant materials/substances, whereas DSs are products containing dietary ingredients such as vitamins, minerals, amino acids, and other dietary substances (e.g., enzymes, organ tissues, glands, metabolites, extracts, and concentrates) [43].

### 2.2. Study Participants

This study included Malaysian individuals who were at least 18 years old, were able to understand written English or Malay language, and had purchased at least one oral HDS that they had never previously used from a community pharmacy in the past three months. Individuals who used HDSs for purposes other than for preventing/treating diseases, or maintaining health were excluded.

### 2.3. Survey Instrument

The survey instrument was developed based on an extensive literature review. Research articles discussing the reasons for consultation with pharmacists around HDS use [40,41,44,45,46,47], pharmacist- or HCP-patient communication and relationship [48,49], and complementary medicine disclosure to HCPs [50,51,52] were used. Based on the literature review, the consultation and non-consultation with CPs about HDSs can be influenced by the consumers’ perceptions of the consultative roles of CPs with respect to HDSs, facilitators (e.g., having a good relationship with CPs, having adequate time to consult CPs, willingness of CPs to spend time providing consultation, etc.), and barriers to consult CPs (e.g., feeling uncomfortable discussing HDSs with CPs, not being asked by CPs about HDS use, not having time to consult CPs, etc.), the health concerns, beliefs of the outcomes of the consultation, and perceived-need of the consultation [40,41,44,45,46,47,48,49].

The survey instrument was initially developed in English and consists of three sections (please see Appendix B). The first section of the questionnaire collected demographic details from the respondents. In this section, the respondents were also asked to indicate the type and name of the HDSs for first time use that they most recently purchased at a community pharmacy. They were then requested to indicate whether or not they had consulted a CP about the HDSs that they had purchased. The second and third sections were dedicated to respondents who consulted and did not consult CPs about their HDS use, respectively. The second section was divided into two parts: reasons for consultation with CPs and topics of discussion during the consultation. The third section also contained two parts: reasons for not consulting CPs and the source of information about the HDSs that the respondents purchased. For sections on the topics of discussion during the consultation with CPs and the source of information about HDSs, the respondents were provided with a list of answer options in which they could select one or more answer options. There were 20 items for the reasons for consultation and non-consultation with CPs, respectively. The respondents were asked to indicate their level of agreement with each item using a 5-point Likert-type scale ranging from 1 = strongly disagree to 5 = strongly agree.

The questionnaire was reviewed by eight academic pharmacists whose working experience ranged from 5 to 20 years. Five of them held a PhD qualification. The purpose of the review was to ensure that each section of the questionnaire and the survey items were relevant and suitable. For the reasons for consultation and non-consultation with CPs, the reviewers were asked to rate the “relevance” of each item by providing a rating of 1 = not relevant; 2 = somewhat relevant; 3 = quite relevant; or 4 = very relevant. They were also requested to rate each item’s “essentiality” (1 = not essential; 2 = useful but not essential; or 3 = essential) and “clarity” (1 = not clear; 2 = item needs some revision; or 3 = very clear). The content validity index (CVI) was calculated for each item based on the reviewers’ ratings. Based on the recommendation by Polit et al., with a reviewer panel consisting of eight individuals, items with a CVI value of ≥0.83 were retained [53].

The content validity study showed that all items had a CVI value of at least 0.83 and therefore were retained in the questionnaire. Furthermore, the mean score for the “essentiality” and “clarity” of the items were acceptable (“essentiality”, range: 2.75 to 3.00; and “clarity”, range: 2.50 to 3.00) (Appendix C). Based on the comments from the reviewers, minor amendments were made to improve the wording and clarity of the items. The questionnaire was then translated into the Malay language by a research team member and back-translated into Malay by another member who was not involved in the survey item development. Subsequently, the back-translated version was compared with the original version, which revealed consistency in meaning.

The questionnaire was then transformed into an online survey using SurveyMonkey, an online service that creates and manages web-based surveys. The online survey was piloted on a convenience sample of 20 individuals to test both the questions and the technical functionality. The pilot test participants included an equal number of consumers who had consulted and not consulted CPs regarding HDSs. The pilot test revealed that the online survey was feasible and the questionnaire was clear. Participants in the pilot test took approximately 10 min to complete the survey. Responses from the respondents in the pilot testing of the questionnaire were not included in the final data analysis of the study.

### 2.4. Data Collection

The present study utilized a convenient, non-probability sampling method in which the link for the survey was distributed to the public using social media applications such as Facebook, WhatsApp, and Telegram. The survey was an “open survey”. The respondents were encouraged to forward the survey to others.

On the introduction page of the online survey, the respondents were informed of the study purpose, the estimated time to complete the survey, that their responses would be both anonymous and confidential, and the list of investigators. The participants were informed that completion of the questionnaire indicated their consent to participate in the study. The following page (screening page) listed several questions to determine the eligibility of the respondents. Those who were not eligible were brought to the last page of the questionnaire to end the survey. Individuals could respond only once to the survey, and no incentive was given for participation.

### 2.5. Statistical Analysis

All statistical analysis was conducted using IBM SPSS version 23 (IBM, Armonk, NY, USA). Continuous data were presented as the mean and standard deviation (SD) whereas categorical data were presented as the frequency and percentage. Univariate and multivariate logistic regression analyses were used to determine the predictors of consultation with CPs regarding HDS use. Odds ratios (ORs) and 95% confidence intervals (CI) were reported for the logistic regression analysis. Statistical significance was established if the *p*-value was <0.05.

## 3. Results

Overall, 1428 individuals responded to the survey and completed the screening questions. Of all the respondents, 642 individuals were not eligible for the study. Among those who were eligible, 193 did not complete the survey, and were therefore removed, resulting in 593 complete responses for the analysis. The completion rate of the survey was 86.5%.

### 3.1. Socio-Demographic Characteristics of Participants (n = 593)

Table 1 shows the socio-demographic characteristics of the study participants. Of all the respondents, most were female (447/593, 75.4%), Malay (557/593, 93.9%), were employed/self-employed (402/593, 67.8%), and earning ≤MYR 2000 monthly (339/593, 57.2%). Participants were predominantly unmarried (362/588, 61.6%), and belonged to the 18–29 age group (332/593, 56%). Approximately half of the participants had a Bachelor’s degree (275/589, 46.7%), and were living in an urban residential setting (308/593, 51.9%). Most participants had no chronic illness (536/593, 90.4%), and were not using any prescription medications (543/593, 91.6%).

Almost 37% (219/593) of the participants indicated that their most recent purchase of HDSs involved herbal products only, whereas about 50% (312/593) indicated that they purchased DSs only. The majority of participants purchased HDSs to maintain health (518/593, 87.4%), whereas 10.5% (62/593), and 18.7% (111/593) intended to use HDSs to prevent and treat disease, respectively. Hypertension (28/593, 4.7%), hyperlipidemia (11/593, 1.9%), and asthma (8/593, 1.3%) were the most common chronic illnesses among the participants (Table 2).

### 3.2. Factors Associated with Consultation with CPs about HDSs (n = 593)

Overall, 40.3% (239/593) of the participants consulted CPs about the HDSs that they purchased. Table 1 shows the comparison of the socio-demographic characteristics of the participants who consulted and did not consult CPs about HDSs. Univariate analysis showed that the individuals who were living in suburban areas were significantly less likely to consult CPs about HDSs compared to those who were living in urban areas (OR = 0.400, 95% CI = 0.269–0.597). Interestingly, individuals who were earning MYR 2001–MYR 5000 were less likely to consult CPs about HDSs compared to those with no monthly income (OR = 0.650, 95% CI = 0.427–0.989). Additionally, the respondents whose most recent purchase involved herbal products only were significantly less likely to consult CPs (OR = 0.667, 95% CI = 0.472–0.942). On the other hand, individuals whose most recent purchase involved DSs only were significantly more likely to consult CPs (OR = 1.496, 95% CI = 1.074–2.084). Multivariate analysis showed that the suburban residential setting was the only significant predictor for consultation with CPs. The adjusted OR of consulting CPs among individuals who were living in suburban areas was lower than those who were living in urban areas (adjusted OR = 0.390, 95% CI = 0.260–0.583) (Table 1).

### 3.3. Reasons for Consulting CPs about HDSs (n = 239)

Table 3 reports the reasons for the participants to consult with CPs about HDSs. The most agreement was indicated for the items: *I felt that the CP was the right person to consult about HDSs* (mean = 4.37, SD = 0.73), *I knew the CP would be willing to discuss my HDS use* (mean = 4.37, SD = 0.67), *I thought the CP was knowledgeable in HDSs* (mean = 4.31, SD = 0.66), *I believe that the CP would understand my reasons for using HDSs* (mean = 4.28, SD = 0.65) and *I believe that the CP was open-minded about my use of HDSs* (mean = 4.24, SD = 0.67). The item with the lowest agreement was *I have a good relationship with the CP* (mean = 3.69, SD = 0.91).

### 3.4. The Topic of Discussion about HDSs during the Consultation with CPs (n = 239)

Among the individuals who consulted CPs about HDSs, the most discussed were the benefits of HDSs (204/239, 85.4%) and the directions for use (178/239, 74.5%). Approximately half discussed the dose (132/239, 55.2%) and side effects (128/239, 53.6%). A minority discussed the potential interactions (65/239, 27.2%) (Table 4).

### 3.5. Reasons for Not Consulting CPs about HDSs (n = 354)

The items with the most agreement among participants who indicated that they did not consult CPs about HDSs were: *I never thought of consulting the CP regarding my use of HDSs* (mean = 3.45, SD = 1.02), *I did not have enough time to consult the CP regarding my use of HDSs* (mean = 3.38, SD = 1.01), *I can make my own decisions regarding my use of HDSs without the help of the CP* (mean = 3.35, SD = 0.92), *The CP did not ask me about my HDS use* (mean = 3.31, SD = 0.96), and *I thought that there was no need to consult the CP because HDSs are safe* (mean = 3.21, SD = 1.03). The items with the lowest agreement were: *I previously had a bad experience when discussing HDSs with a CP* (mean = 2.09, SD = 0.94), *I thought the CP did not know about HDSs* (mean = 2.21, SD = 0.97), and *I felt that the CP was not the right person to consult about HDSs* (mean = 2.32, SD = 1.04) (Table 5).

### 3.6. Sources of Information about HDSs among Respondents Who Did Not Consult CPs about HDSs (n = 354)

Among the individuals who did not consult CPs about HDSs, the majority referred to the Internet (217/354, 61.3%) and social media (212/354, 59.9%) for HDS information. Approximately, 40% of them referred to their family members (144/354, 40.7%) and friends (132/354, 37.3%). A minority of participants referred to doctors (40/354, 11.3%) and nutritionists (40/354, 11.3%). Research articles were the least preferred source of information about HDSs among the participants (3/354, 0.8%) (Table 6).

## 4. Discussion

This study was the first to examine the prevalence of consultation with CPs about HDSs among a sample of the public who had purchased HDSs for first time use at a community pharmacy. It showed that the prevalence with which HDS consumers consulted CPs about HDSs was low, even though the products were purchased at the pharmacy. In the present study, out of 593 respondents, only 40.3% (239/593) consulted the CPs about the HDSs they purchased, meaning that 59.7% (354/593) did not. Our findings may assist health authorities, pharmacy professional bodies, and researchers in Malaysia and other countries to develop strategies to promote consultation with CPs among HDS consumers.

In this study, most HDS consumers who consulted with CPs agreed that these were the right people to discuss HDSs with, and that CPs were willing to be consulted about HDSs. Our findings are encouraging as they show that consumers viewed CPs as consultants for HDSs. In a previous focus group study by Kwan et al., consumers in Canada had similar opinions. In this regard, consumers were willing to seek partnerships with pharmacists around decision-making concerning HDS use to ensure the safe use of the products. However, it should be noted that in the study, the consumers believed that the final decision to use HDSs, or otherwise, should not be dictated by the pharmacists [41]. This reflects an interpretive model of the patient–HCP relationship in which pharmacists provide consumers with information and assist them in elucidating and articulating their values, but do not participate directly in the decision-making [49].

In the present study, consumers who consulted the CPs wanted them to be actively involved in their decision-making regarding their HDS use. This reflects a model of the patient–HCP relationship that is consistent with the deliberative model. In this model, both consumers and CPs actively participate in a decision-making process through exchanges of information [49]. This is supported by our findings, which showed that the majority of the consumers who consulted the CPs wanted them to provide information (89.5%, 214/239), give advice (85.8%, 205/239), help in decision-making (82.8%, 198/239), and approve their HDS use (74.9%, 179/239).

Other noteworthy findings in this study are that the majority of respondents who consulted CPs agreed that CPs are knowledgeable about HDSs and are able to provide trustworthy HDS information. Multiple studies have reported that pharmacists are considered a major source of reliable HDS information among HDS consumers. Despite this, doubts about the extent of the pharmacists’ knowledge about HDSs among consumers have been reported. For example, in the study by Kwan et al., participants believed that some pharmacists were not knowledgeable about HDSs and expressed concerns about their ability as gatekeepers to consumer safety [41]. In a study in Lebanon, Hijazi et al., reported that the majority of consumers (61.3%) did not regard CPs as more knowledgeable about HDSs than other HCPs [46]. Furthermore, in previous studies involving pharmacists in various countries such as the U.S., Australia, Thailand, Singapore, and Malaysia, many pharmacists have been reported to have inadequate knowledge about HDSs [34,40,54,55,56,57]. Thus, the high agreement in this study regarding CPs’ expertise in HDSs among individuals who consulted them is greatly encouraging. The finding shows that there is a positive professional image of CPs as consultants for HDS use from the consumers’ perspective. This may have resulted from the increased interest in HDSs among CPs in Malaysia in recent years, as evidenced by many CPs offering health supplement consultation services at pharmacies, and completing Complementary Medicine Education (CMed) programs endorsed by local pharmacy organizations [58,59].

That being said, about 60% of the present study’s respondents did not consult the CPs about HDSs. This implies that there is still a lack of recognition and awareness of the roles of CPs related to HDSs among the public, and shows that CPs are still underutilized for information on HDSs. This is further supported by our findings, showing that more than half of the respondents who did not consult CPs about their HDS use had never thought of doing so (54.2%, 192/354), and about 40% (153/354) felt that there was no need to consult CPs because HDSs are safe. Another concern that arose from our study was that respondents who are more at risk of HDS-related problems such as older people, consumers with chronic diseases, and those who were using conventional medicines were not significantly more likely to consult CPs about their HDS use. It is noteworthy that some HDSs may interact with conventional medicines. For example, vitamin E may increase the bleeding risk of warfarin, black cohosh may reduce the effectiveness of statins, and St. John’s wort can reduce the effectiveness of various drugs such as cyclosporine, warfarin, theophylline, and digoxin [60]. Thus, a consultation with a CP about HDSs is important, especially for those who are using conventional medicines. This would allow CPs to check for HDS–drug interactions and prevent potential interactions [33].

Additionally, we observed that individuals living in suburban areas were less likely to consult CPs about HDSs than those living in urban areas. This is a concern because in Malaysia, HDSs are prevalently used among those living in suburban areas [1,61,62,63]. While no data can be compared directly to our result, in a study conducted in Michigan, USA, Malewski et al., reported that individuals who lived in a suburban area were less likely to seek advice from CPs about medications than those who lived in an urban area [64]. Although not confirmed in this study, the characteristics of suburban pharmacies and their predominant activities or services may influence the consumers’ consulting behavior with CPs. Of note, previous studies have reported fewer offerings of pharmaceutical care services, fewer opportunities to consult CPs, and higher prescription volumes at suburban pharmacies [65,66,67].

Our findings warrant more efforts from the government and professional pharmacy bodies to encourage consumers to discuss their HDS use with pharmacists. Campaigns such as “Know Your Medicine” and “Know Your Pharmacists”, which have been widely employed in Malaysia and other parts of the world, can be leveraged by highlighting the role of CPs in ensuring the safe use of HDSs [68]. These campaigns can be targeted toward people in suburban areas and those who are more at risk of HDS-related issues (e.g., older people, consumers with chronic diseases, and prescription medication users). Moreover, it is worth highlighting that in the present study, among those who consulted CPs, their discussion about HDSs mostly revolved around the benefits and directions for use, but little on the potential risks (e.g., side effects and HDS–drug interactions). Therefore, the public should be educated on the importance of being aware of the potential risks of HDS use. Most importantly, they should be encouraged to discuss both the benefits and risks of HDS use with a HCP.

In this study, about half of the individuals who did not consult CPs about their HDS use (51.4%, 182/354) indicated that they had no time for the consultation. In a study in Thailand, Wahab et al., reported that the unwillingness of consumers to spend time consulting CPs was a barrier to the latter performing their professional duties around HDS use [40]. CPs in the study characterized those consumers as “pick-and-go” customers who normally avoided consultation with CPs [40]. This caused CPs to avoid engaging with them, meaning they were left to decide about HDS use on their own. Additionally, almost half of the individuals who did not consult CPs (49.4%, 175/354) believed that they could make their own decisions regarding HDS use, whereas 40.4% (143/354) believed that they were well-informed about the HDSs that they purchased. These consumers fit the characteristics of “new consumers” or “lay experts”, as described in previous studies [40,41,48].

The “new consumers” are individuals who seek independence and autonomy in their self-care [69]. This type of consumer frequently utilizes a variety of resources to learn about their self-care needs [70] and regularly perceives that they have adequate knowledge to decide on HDS use [40]. The “lay experts” usually exhibit a strong sense of perceived ability for self-care and often refuse to obtain the pharmacist’s advice [40,48]. Nonetheless, although many of the respondents who did not consult CPs were confident in their ability to decide on their HDS use, or perceived themselves as knowledgeable about the products, our results showed that the majority of them referred to the Internet (61.3%, 217/354) and social media (59.9%, 212/354) for information about HDSs.

The fact that those who regarded themselves as well-informed about HDSs reported using information from the Internet and social media is less than reassuring, since information obtained from these sources can be of poor quality, misleading, and anecdotal [71,72]. For example, previous studies assessing websites on herbal products showed that the majority were low quality, with most not containing information on the HDS–drug interactions, contraindications, and adverse effects [71] as well as containing biased information [73]. Furthermore, a content analysis study on social media claims about HDSs revealed that almost all claims identified for HDSs were “potentially misleading”, with the majority tending to exaggerate the efficacy or safety without sufficient evidence [72]. Thus, in addition to encouraging HDS consumers to consult pharmacists regarding the use of HDSs, efforts to raise the awareness of Internet and social media users of how to identify good quality HDS information are warranted.

## 5. Strengths and Limitations of the Study

This study had several limitations. First, since was a cross-sectional study, it only provides a snapshot of the participants’ responses at the time of the survey. Additionally, since this study distributed the survey mainly through social media, there is a possibility that individuals who infrequently use digital technology (e.g., older people and those in remote areas [74]) were left out of the survey. Furthermore, since the data collected in this survey was self-reported, under- or over-reporting of information is possible. Additionally, considering that the study sought the consumers’ responses on their consultation with CPs during the COVID-19 pandemic, their consultation or non-consultation with CPs may have been influenced by other situational factors related to the pandemic (e.g., large queues in the pharmacy, or a reluctance to spend a long time in the pharmacy) that were not captured in this study. Moreover, our sample only included a small proportion of individuals that were considered “high-risk” for HDS use such as older people, people with multiple comorbidities, and those using prescription medications. Future studies should focus on these populations. Additionally, because our sample was composed mostly of healthy individuals, their health state may have impacted the perceived need to consult CPs about HDSs. Finally, since community pharmacy practice and consumer behavior may vary in other countries, the prevalence and factors of consultation with CPs about HDSs may be different in other countries.

Nevertheless, the present study provides valuable insights into the prevalence of as well as the reasons for consultation or non-consultation with CPs about HDSs among consumers in Malaysia. The incorporation of wide-ranging reasons for consultation or non-consultation with CPs about HDSs in the survey instrument allowed for a comprehensive understanding of the topic and can inform strategies to further encourage consumers to consult CPs. The data obtained from this study can benefit the Malaysian health authorities, pharmacy professional bodies, and researchers by serving as baseline information for future research, or interventional programs.

## 6. Conclusions

In conclusion, less than half of the consumers consulted CPs about their HDS use. The individuals who did consult CPs were generally positive about the roles of CPs with respect to HDSs and wanted them to participate in decision-making around the use of the products. Meanwhile, individuals who did not consult CPs appeared to be unaware of the roles of CPs in ensuring the safe use of HDSs. Additionally, they mostly believed that they were able to make their own decisions around HDS use, and perceived themselves as knowledgeable about HDSs. Despite this, most of those who did not consult CPs referred to the Internet and social media for HDS information. The findings from this study warrant more efforts from the government, pharmacy professional bodies, and HCPs to encourage consumers to consult pharmacists about their HDS use and to enhance their awareness of the role of CPs in ensuring the safe use of HDSs.

## Figures and Tables

**Table 1:** The socio-demographic characteristics of the study respondents and comparison of the socio-demographic characteristics among those who consulted and did not consult CPs regarding HDSs (*n* = 593).

Characteristic		All (*n* = 593) ^a^	Consulted CP (*n* = 239)	Did Not Consult CP (*n* = 354)	Univariate		Multivariate	
					Odds Ratio (95% CI)	*p*-Value	Odds Ratio (95% CI)	*p*-Value
**Gender**	Female	447 (75.4)	183 (76.6)	264 (74.6)	Reference	-		
	Male	146 (24.6)	56 (23.4)	90 (25.4)	0.898 (0.612–1.317)	0.581		
**Marital Status (*n* = 588) ^b^**	Unmarried	362 (61.6)	155 (65.4)	207 (59)	Reference	-		
	Married	226 (38.4)	82 (34.6)	144 (41)	0.760 (0.540–1.070)	0.116		
**Ethnicity**	Malays	557 (93.9)	220 (92.1)	337 (95.2)	Reference	-		
	Non-Malay	36 (6.1)	19 (7.9)	17 (4.8)	1.712 (0.871–3.366)	0.119		
**Age group**	18–29 years	332 (56)	146 (61.1)	186 (52.5)	Reference	-		
	30–39 years	117 (19.7)	42 (17.6)	75 (21.2)	0.713 (0.462–1.103)	0.129		
	40–49 years	96 (16.2)	31 (13)	65 (18.4)	0.608 (0.376–0.981)	0.420		
	50 years above	48 (8.1)	20 (8.4)	28 (7.9)	0.910 (0.493–1.680)	0.763		
**Highest education level (*n* = 589) ^c^**	Diploma and below	164 (27.8)	61 (25.6)	103 (29.3)	Reference	-		
	Bachelor’s degree	275 (46.7)	123 (51.7)	152 (43.3)	1.366 (0.920–2.030)	0.122		
	Postgraduate degree	150 (25.5)	54 (22.7)	96 (27.4)	0.950 (0.600–1.504)	0.826		
**Type of residential setting**	Urban	308 (51.9)	146 (61.1)	162 (45.8)	Reference	-	Reference	-
	Suburban	181 (30.5)	48 (20.1)	133 (37.6)	0.400 (0.269–0.597)	<0.001	0.390 (0.260–0.583)	<0.001
	Rural	104 (17.5)	45 (18.8)	59 (16.7)	0.846 (0.541–1.324)	0.456	0.731 (0.458–1.165)	0.187
**Employment status**	Employed/self-employed	402 (67.8)	153 (64)	249 (70.3)	Reference	-		
	Students and unemployed	191 (32.2)	86 (36)	105 (29.7)	0.750 (0.529–1.064)	0.107		
**Monthly income**	No income	174 (29.3)	80 (33.5)	94 (26.6)	Reference	-	Reference	-
	MYR 2000 and lower	80 (13.5)	35 (14.6)	45 (12.7)	0.914 (0.536–1.557)	0.741	0.958 (0.555–1.165)	0.879
	MYR 2001–MYR 5000	191 (32.2)	68 (28.5)	123 (34.7)	0.650 (0.427–0.989)	0.044	0.702 (0.454–1.087)	0.113
	More than MYR 5000	148 (25)	56 (23.4)	92 (26)	0.715 (0.458–1.118)	0.141	0.779 (0.487–1.244)	0.295
**Chronic illnesses**	Yes	57 (9.6)	27 (11.3)	30 (8.5)	Reference	-		
	No	536 (90.4)	212 (88.7)	324 (91.5)	0.727 (0.420–1.258)	0.254		
**Use prescription medications**	Yes	50 (8.4)	25 (10.5)	25 (7.1)	Reference	-		
	No	543 (91.6)	214 (89.5)	329 (92.9)	0.650 (0.364–1.162)	0.146		
**Most recent purchase involved herbal product only**	Yes	219 (36.9)	75 (31.4)	144 (40.7)	0.667 (0.472–0.942)	0.022	0.754 (0.415–1.372)	0.356
	No	374 (63.1)	164 (68.6)	210 (59.3)	Reference	-	-	-
**Most recent purchase involved dietary supplement only**	Yes	312 (52.6)	140 (58.6)	172 (48.6)	1.496 (1.074–2.084)	0.017	1.169 (0.656–2.081)	0.596
	No	281 (47.4)	99 (41.4)	182 (51.4)	Reference	-	-	-
**Most recent purchase involved both herbal product and dietary supplement**	Yes	62 (10.5)	24 (10)	38 (10.7)	0.928 (0.541–1.592)	0.787		
	No	531 (89.5)	215 (90)	316 (89.3)	Reference	-		
**HDS purchased was intended to prevent disease**	Yes	173 (29.2)	69 (28.9)	104 (29.4)	0.976 (0.680–1.400)	0.976		
	No	420 (70.8)	170 (71.1)	250 (70.6)	Reference	-		
**HDS purchased was intended to treat disease**	Yes	111 (18.7)	47 (19.7)	64 (18.1)	1.109 (0.730–1.685)	0.627		
	No	482 (81.3)	192 (80.3)	290 (81.9)	Reference	-		
**HDS purchased was intended to maintain health**	Yes	518 (87.4)	215 (90)	303 (85.6)	1.508 (0.900–2.525)	0.119		
	No	75 (12.6)	24 (10)	51 (14.4)	Reference	-		

CP—community pharmacist; CI—confidence interval; HDS—herbal and dietary supplement; MYR—Malaysian Ringgit. ^a^ Unless stated otherwise. ^b^ Five respondents “preferred not to answer”. ^c^ Four respondents “preferred not to answer”.

**Table 2:** The underlying medical conditions among the respondents (*n* = 593).

Underlying Medical Condition	*n* (%)
Hypertension	28 (4.7)
Hyperlipidemia	11 (1.9)
Asthma	8 (1.3)
Skin disease	6 (1)
Heart disease	3 (0.5)
Arthritis	2 (0.3)
Osteoporosis	2 (0.3)
Thyroid disease	2 (0.3)
Stroke	1 (0.2)
Systemic lupus erythematosus	1 (0.2)
Gastritis	1 (0.2)
Celiac disease	1 (0.2)
Weakened immune system	1 (0.2)
Cancer	1 (0.2)
Migraine	1 (0.2)
Renal disease	1 (0.2)
Endometriosis	1 (0.2)
Hypersensitivity	1 (0.2)

**Table 3:** The reasons for consulting CPs about HDSs (*n* = 239).

Reasons for Consulting CPs about HDSs	Mean Score	Strongly Disagree/Disagree	Unsure	Agree/Strongly Agree
1. I knew the CP would be willing to discuss my HDS use	4.37 ± 0.67	1 (0.4)	22 (9.2)	216 (90.4)
2. I felt that the CP was the right person to consult about HDSs	4.37 ± 0.73	2 (0.8)	30 (12.6)	207 (89.6)
3. I thought the CP was knowledgeable in HDSs	4.31 ± 0.66	1 (0.4)	20 (8.4)	218 (91.2)
4. I believe that the CP would understand my reasons for using HDSs	4.28 ± 0.65	2 (0.8)	20 (8.4)	217 (90.8)
5. I believe that the CP was open-minded about my use of HDSs	4.24 ± 0.67	1 (0.4)	28 (11.7)	210 (87.9)
6. I knew the CP would be able to provide me with trustworthy information about HDSs	4.22 ± 0.68	3 (1.2)	22 (9.2)	214 (89.5)
7. I felt that the CP could help me in selecting appropriate HDSs	4.20 ± 0.63	1 (0.4)	25 (10.5)	213 (89.1)
8. I believe the CP would be concerned about my well-being	4.20 ± 0.71	0 (0)	40 (16.7)	199 (83.3)
9. I knew the CP had good opinions about HDSs	4.18 ± 0.69	2 (0.8)	32 (13.4)	205 (85.8)
10. I wanted the CP’s advice about HDSs	4.18 ± 0.79	7 (3)	27 (11.3)	205 (85.8)
11. I felt that the CP could help me in making decisions about the use of HDSs	4.16 ± 0.72	2 (0.8)	39 (16.3)	198 (82.8)
12. I knew the CP would let me decide about my use of HDSs as long as it would not cause harm	4.13 ± 0.71	4 (1.6)	29 (12.1)	206 (86.2)
13. The CP was willing to spend time discussing my HDS use	4.08 ± 0.74	4 (1.7)	41 (17.2)	194 (81.2)
14. I felt comfortable discussing HDSs with the CP	4.07 ± 0.74	3 (1.3)	48 (20.1)	188 (78.7)
15. I was concerned about the side effects of the HDS I was using	4.00 ± 0.90	15 (6.3)	46 (19.2)	178 (74.5)
16. I knew the CP would support my use of HDSs	3.97 ± 0.74	3 (1.2)	57 (23.8)	179 (74.9)
17. I wanted the CP’s approval of my HDS use	3.97 ± 0.79	9 (1.5)	51 (21.3)	179 (74.9)
18. The CP asked me about my use of HDSs	3.91 ± 0.84	16 (6.7)	44 (18.4)	179 (74.9)
19. I was concerned about drug interactions with the HDS I was using	3.90 ± 1.06	26 (10.9)	38 (15.9)	175 (73.2)
20. I have a good relationship with the CP	3.69 ± 0.91	14 (5.8)	102 (42.7)	123 (51.5)

CP—community pharmacist; HDSs—herbal and dietary supplements.

**Table 4:** The topic of discussion about HDSs during the consultation with CPs (*n* = 239).

Topic of Discussion	*n* (%)
Benefits	204 (85.4)
Direction for use	178 (74.5)
Dose	132 (55.2)
Side effects	128 (53.6)
Indication	99 (41.4)
Potential interaction with medications or other HDSs	65 (27.2)
Do not remember	8 (3.3)

HDSs—herbal and dietary supplements.

**Table 5:** The reasons for not consulting CPs about HDSs (*n* = 354).

Reasons for Not Consulting CPs about HDSs	Mean Score	Strongly Disagree/Disagree	Unsure	Agree/Strongly Agree
1. I never thought of consulting the CP regarding my use of HDSs	3.45 ± 1.02	64 (18.1)	98 (27.7)	192 (54.2)
2. I did not have enough time to consult the CP regarding my use of HDSs	3.38 ± 1.01	70 (19.8)	102 (28.8)	182 (51.4)
3. I can make my own decision regarding my use of HDSs without the help of the CP	3.35 ± 0.92	60 (16.9)	119 (33.6)	175 (49.4)
4. The CP did not ask me about my HDS use	3.31 ± 0.96	66 (18.6)	134 (37.9)	154 (43.5)
5. I thought that there was no need to consult the CP because HDSs are safe	3.21 ± 1.03	92 (26)	109 (30.8)	153 (43.2)
6. I am well-informed about HDSs	3.20 ± 0.93	82 (23)	129 (36.4)	143 (40.4)
7. I did not like to talk to the CP regarding my use of HDSs	2.94 ± 1.07	130 (36.7)	114 (32.2)	110 (31.1)
8. I felt uncomfortable discussing HDSs with the CP	2.86 ± 1.06	147 (41.5)	97 (27.4)	110 (31.1)
9. I believed consultation about my use of HDSs with the CP was not necessary	2.85 ± 0.93	139 (39.3)	121 (34.2)	94 (26.6)
10. I was worried the CP would not support my use of HDSs	2.77 ± 1.05	147 (41.5)	113 (31.9)	94 (26.6)
11. I was worried the CP would respond negatively about my HDS use	2.76 ± 1.06	149 (42.1)	117 (33.1)	88 (24.9)
12. I knew the CP would not support my use of HDSs	2.63 ± 1.03	161 (67.4)	127 (35.9)	66 (18.6)
13. I thought the CP would not understand my choice in using HDSs	2.54 ± 0.97	187 (52.8)	93 (26.3)	74 (12.5)
14. I knew the CP had bad opinions about HDSs	2.54 ± 0.95	170 (48)	137 (38.7)	47 (12.2)
15. I thought that the CP was not open-minded about my use of HDSs	2.51 ± 1.06	193 (54.5)	99 (28)	62 (17.5)
16. I thought that the CP was not willing to discuss my HDS use	2.51 ± 1.03	194 (54.8)	99 (28)	61 (17.2)
17. I found it hard to accept opinions from CPs about HDSs	2.34 ± 0.91	225 (63.6)	94 (26.6)	35 (9.9)
18. I felt that the CP was not the right person to consult about HDSs	2.32 ± 1.04	215 (60.7)	93 (26.3)	46 (13)
19. I thought the CP did not know about HDSs	2.21 ± 0.97	244 (68.9)	76 (21.5)	34 (9.6)
20. I previously had a bad experience when discussing HDSs with a CP	2.09 ± 0.94	249 (70.3)	78 (22)	27 (7.6)

CP—community pharmacist; HDSs—herbal and dietary supplements.

**Table 6:** Sources of information about HDSs among the respondents who did not consult CPs about HDSs (*n* = 354).

Source of Information	*n* (%)
Internet	217 (61.3)
Social media	212 (59.9)
Family members	144 (40.7)
Friends	132 (37.3)
Nutritionists	40 (11.3)
Doctors	40 (11.3)
Television	30 (8.5)
Radio	6 (1.7)
Religious texts	6 (1.7)
Research articles	3 (0.8)

## Data Availability

The data presented in this study are available on request from the corresponding author. The data are not publicly available due to privacy.

## Appendix A

**Table A1:** Reporting of the study procedures and results according to the Checklist for Reporting Results of Internet E-Surveys (CHERRIES).

Item Category	Checklist Item	Description
Design	Study design	This was a cross-sectional study that was conducted over three weeks from 4 to 25 April 2022 involving conveniently sampled HDS consumers in Malaysia.
Ethics	Institutional Review Board (IRB) approval	This study was approved by the Research Ethics Committee of *Universiti Teknologi* MARA (UiTM), Malaysia (REC[PH]/014/2022).
	Informed consent	On the introduction page of the online survey, the respondents were informed of the study purpose, the estimated duration to complete the survey, that the data would be only used for the study, and the list of investigators. They were also informed that their participation in the study would be voluntary, and they were offered anonymity and confidentiality. Completion of the questionnaire indicated their consent to participate in the study.
	Data protection	No personal-identifying information was collected.
Development and pre-testing		The survey instrument was developed based on an extensive literature review. Research articles discussing the reasons for consultation with pharmacists around HDS use, and relevant studies on pharmacist– or HCP–patient communication and relationship, and the literature on CM disclosure to HCPs were also referred. For content validity, the questionnaire was reviewed by eight academic pharmacists whose working experienced ranged from 5 to 20 years. The content validity study showed that the items in the questionnaire were relevant, essential, and clear. Subsequently, the questionnaire was piloted on 20 individuals. The pilot test revealed that the questionnaire was clear and took approximately 10 min to complete.
Recruitment process	Open vs. closed survey	The data were collected using an open survey.
	Contact mode	The link for the survey was distributed to the public using social media applications such as Facebook, WhatsApp, and Telegram. The respondents were free to fill in the questionnaire and encouraged to forward the survey to others.
	Advertising the survey	The survey was not advertised.
Survey administration	Web/email	The survey was managed using SurveyMonkey, an online service that creates and manages web-based surveys.
	Context	N/A.
	Mandatory/voluntary	The participation of the respondents in the study was voluntary, and they were offered anonymity and confidentiality.
	Incentives	None.
	Time/Date	4–25 April 2022
	Randomization of items or questionnaire	N/A.
	Adaptive questioning	Adaptive questioning (branched) was used. Relevant survey items were displayed based on previous responses (e.g., only those who reported that they consulted CPs about their HDS use were shown the follow-up questions about the reasons for the consultation).
	Number of items	The full survey comprised a total of 56 items, although because of the adaptive nature of the questionnaire, not all respondents answered all items.
	Number of screens (pages)	Five pages.
	Completeness check	The respondents were required to complete mandatory questions before proceeding to the next page.
	Review step	Participants could use a “Back” button.
Response rates	Unique site visitor	N/A.
	View rate	N/A.
	Participation rate	Overall, 1428 individuals responded to the survey and completed the screening questions. Of all the individuals, 642 individuals were not eligible for the study. Among those who were eligible, 193 did not complete the survey and were therefore removed, resulting in 593 complete responses for the analysis.
	Complete rate	The completion rate of the survey was 86.5%.
Preventing multiple entries	Cookies used	Cookies were not used.
	IP check	IP addresses were collected by the survey administration tool (SurveyMonkey).
	Log file analysis	The study did not include a log file analysis.
	Registration	N/A.
Analysis	Handling of incomplete questionnaires	Respondents with an incomplete questionnaire were removed (*n* = 193)
	Questionnaires submitted with an atypical timestamp	N/A.
	Statistical correction	N/A.

HDS—herbal and dietary supplement; CPs—community pharmacists; N/A—non-applicable [42].

## Appendix B

**Table A2:** Structure and Content of the Questionnaire.

Section/Page	Theme	Content	Number of Questions
1 (Introduction page)	-	The participants were informed on the introductory page of the online survey that the survey was concerned with their consultation with CPs regarding HDSs; that it would take approximately 10 min to complete; that all responses were confidential and anonymous; and that reporting would be on an aggregate level only. Consent was indicated when respondents clicked on the ‘Next’ button from this page.	
2 (Screening page)	-	The screening page lists several criteria to determine the eligibility of the respondents in the study (being a Malaysian citizen, age ≥18 years old, able to understand written English or Malay language, had purchased at least one type of oral HDS for first time use from a community pharmacy in the past three months, and the purpose of the HDS(s) purchased was/were to prevent and/or treat diseases or to maintain health).	5
3 ^a^	Socio-demographic characteristics, history of chronic illness and medication use, and characteristics of HDSs purchased	1. Gender (Female/Male) 2. Marital status (Single/Married/Divorced/Widow or Widower) 3. Ethnicity (Malay/Chinese/Indian/Others) 4. Age group (18–29 years/30–39 years/40–49 years/≥50 years) 5. Highest education level (No formal education/Primary or secondary education/Certificate/Diploma/Bachelor’s degree/Master’s degree or PhD) 6. Type of residential setting (Urban/Suburban/Rural) 7. Employment status (Employed or Self-employed/Unemployed/Students) 8. Monthly income (No income/MYR 2000 and Lower/MYR 2001–MYR 5000/More than MYR 5000) 9. History of chronic illness (Yes/No) 10. Type of chronic illness (Diabetes/Hypertension/Hyperlipidemia/Asthma/Heart Disease/Others) ^b^ 11. History of taking prescription medication (Yes/No) 12. Category and name of HDSs purchased (Herbal Product/Dietary Supplement/Both) 13. Purpose of use of the HDSs purchased (To Prevent Disease/To Treat Disease/To Maintain Health) 14. History of consultation with CPs about the HDSs purchased (Yes/No)	14
4 ^c^	Reasons for consulting CPs about HDSs and topic of discussion	1. Reasons for consulting CPs about HDSs (Please see Appendix C: Table A3) 2. Topic of discussion about HDSs during the consultation with CPs (Benefits/Direction for Use/Dose/Side Effects/Indication/Potential Interactions with Medications or Other HDSs/Do Not Remember/Others) ^b^	20 1
5 ^d^	Reasons for not consulting CPs about HDSs and source of information about HDSs	1. Reasons for not consulting CPs about HDSs (Please see Appendix C: Table A4) 2. Source of information about HDSs (Internet/Social Media/Family Members/Friends/Nutritionists/Doctors/Television/Radio/Others) ^b^	20 1

CPs—community pharmacists; HDSs—herbal and dietary supplements. ^a^ To be answered by all respondents. ^b^ A list of answer options are provided. The respondents may pick the response option “others” if their answer(s) is/are not included in the provided list. They may then indicate their answer(s) in the comment section. ^c^ To be answered by respondents who consulted CPs about HDSs. ^d^ To be answered by respondents who did not consult CPs about HDSs.

## Appendix C

**Table A3:** Content Validity Index (CVI), and Mean “Essentiality” and “Clarity” Scores of “Reasons for Consulting CPs about HDS Use” Items.

Items	CVI	Essentiality Mean Score	Clarity Mean Score
1. I felt that the CP was the right person to consult about HDSs	1.00	3.00	3.00
2. I knew the CP would be willing to discuss my HDS use	1.00	3.00	2.88
3. I believe the CP would be concerned about my well-being	1.00	2.88	2.75
4. I believe that the CP would understand my reasons for using HDSs	0.88	2.75	2.75
5. I thought the CP was knowledgeable in HDSs	1.00	2.88	2.88
6. I believe that the CP was open-minded about my use of HDSs	0.88	2.86	2.57
7. I knew the CP would support my use of HDSs	0.88	2.88	2.88
8. I knew the CP had good opinions about HDSs	0.88	2.88	2.63
9. I have a good relationship with the CP	1.00	2.88	2.75
10. I felt comfortable discussing HDSs with the CP	1.00	3.00	3.00
11. The CP asked me about my use of HDSs	0.88	3.00	2.88
12. The CP was willing to spend time discussing my HDS use	0.88	3.00	3.00
13. I wanted the CP’s approval of my HDS use	1.00	3.00	2.63
14. I was concerned about the side effects of the HDS I was using	1.00	3.00	2.63
15. I was concerned about drug interactions with the HDS I was using	1.00	3.00	2.88
16. I wanted the CP’s advice about HDSs	1.00	3.00	3.00
17. I felt that the CP could help me in making decisions about the use of HDSs	1.00	3.00	2.88
18. I felt that the CP could help me in selecting appropriate HDSs	1.00	3.00	3.00
19. I knew the CP would let me decide about my use of HDS as long as it would not cause harm	1.00	3.00	2.75
20. I knew the CP would be able to provide me with trustworthy information about HDSs	1.00	3.00	3.00

HDSs—herbal and dietary supplements.

**Table A4:** Content Validity Index (CVI), and Mean “Essentiality” and “Clarity” Scores of “Reasons for Not Consulting CPs about HDS Use” Items.

Items	CVI	Essentiality Mean Score	Clarity Mean Score
1. I felt that the CP was not the right person to consult about HDSs	1.00	3.00	3.00
2. I thought that the CP was not willing to discuss my HDS use	1.00	3.00	2.88
3. I thought the CP would not understand my choice in using HDSs	1.00	3.00	2.75
4. I thought the CP did not know about HDSs	1.00	3.00	2.88
5. I thought that the CP was not open-minded about my use of HDSs	0.88	3.00	2.88
6. I knew the CP would not support my use of HDSs	0.88	2.88	2.88
7. I knew the CP had bad opinions about HDSs	1.00	2.88	2.63
8. I felt uncomfortable discussing HDSs with the CP	1.00	3.00	2.50
9. The CP did not ask me about my HDS use	1.00	3.00	3.00
10. I did not like to talk to the CP regarding my use of HDSs	1.00	3.00	3.00
11. I found it hard to accept opinions from CPs about HDSs	1.00	3.00	3.00
12. I did not have enough time to consult the CP regarding my use of HDSs	1.00	3.00	3.00
13. I never thought of consulting the CP regarding my use of HDSs	1.00	3.00	3.00
14. I thought that there was no need to consult the CP because HDSs are safe	1.00	3.00	3.00
15. I can make my own decision regarding my use of HDS without the help of the CP	1.00	3.00	2.88
16. I am well-informed about HDSs	1.00	3.00	3.00
17. I believed consultation about my use of HDS with the CP was not necessary	1.00	2.88	3.00
18. I previously had a bad experience when discussing HDS with a CP	1.00	3.00	3.00
19. I was worried the CP would not support my use of HDSs	0.88	3.00	3.00
20. I was worried the CP would respond negatively about my HDS use	0.88	2.88	3.00

CP—community pharmacist; HDSs—herbal and dietary supplements.
